# TinoTranscriptDB: A Database of Transcripts and Microsatellite Markers of *Tinospora cordifolia*, an Important Medicinal Plant

**DOI:** 10.3390/genes13081433

**Published:** 2022-08-12

**Authors:** Rakesh Singh, Ajay Kumar Mahato, Akshay Singh, Rajesh Kumar, Amit K. Singh, Sundeep Kumar, Soma S. Marla, Ashok Kumar, Nagendra K. Singh

**Affiliations:** 1 ICAR-National Bureau of Plant Genetic Resources, Pusa, New Delhi 110012, India; 2 The Centre for DNA Fingerprinting and Diagnostics, Hyderabad 500039, India; 3 ICAR-National Institute for Plant Biotechnology, Pusa, New Delhi 110012, India

**Keywords:** *Tinospora*, markers, simple sequence repeats (SSRs), transcriptome, gene ontology (GO), transcription factors (TFs)

## Abstract

*Tinospora cordifolia*, commonly known as “Giloe” in India, is a shrub belonging to the family Menispermaceae. It is an important medicinal plant known for its antipyretic, anti-inflammatory, antispasmodic, and antidiabetic properties and is used in the treatment of jaundice, gout, and rheumatism. Despite its economic importance, the limited information related to its genomic resources prohibits its judicious exploitation through molecular breeding or biotechnological approaches. In this study, we generated a meta-transcriptome assembly of 43,090 non-redundant transcripts by merging the RNASeq data obtained from Roche 454 GS-FLX, and Illumina platforms, and report the first transcriptome-based database for simple sequence repeats and transcription factors (“TinoTranscriptDB” (*Tinospora cordifolia* Transcriptome Database)). We annotated 26,716 (62%) of the total transcripts successfully from National Center for Biotechnology Information non-redundant protein (NCBI-NR), gene ontology (GO), Kyoto Encyclopedia of Genes and Genomes (KEGG), Swiss-Prot, and Pfam databases. This database contains information of 2620 perfect simple sequence repeats (P-SSRs) with a relative abundance of 340.12 (loci/Mb), and relative density of 6309.29 (bp/Mb). Excluding mono-nucleotides, the most abundant SSR motifs were tri-nucleotides (54.31%), followed by di-nucleotides (37.51%), tetra-nucleotides (4.54%), penta-nucleotides (3.16%) and hexa-nucleotides (0.45%). Additionally, we also identified 4,311 transcription factors (TFs) and categorized them into 55 sub-families. This database is expected to fill the gap in genomic resource availability in *T. cordifolia* and thus accelerate molecular breeding and related functional and other applied studies aimed towards genetic improvements of *T. cordifolia* and related species.

## 1. Introduction

*T. cordifolia* (Willd.) Miers., commonly named as “Giloe” or “Guduchi”, is a large, perennial, deciduous climbing shrub belonging to the family Menispermaceae. It is distributed throughout the tropical Indian sub-continent, Myanmar, Sri Lanka, and China [1]. A wide range of active ingredients such as alkaloids, steroids, diterpenoids, lactones, glycosides, sesquiterpenoids, phenolics, polysaccharides, and aliphatic compounds have been isolated from the different parts of the plant, including, stem, root, and whole plant [2,3]. *T. cordifolia* is known for its antispasmodic, anti-inflammatory, antidiabetic, antiallergic, antipyretic, and analgesic properties and is being used in the treatment of gout, jaundice, rheumatism, urinary disorder, diabetes, anemia, skin diseases and infections [4,5,6,7]. According to the Ayurveda (Indian medicine) literature, the phytoactive ingredients of *T. cordifolia* are to a constituent of several formulations which is used for the treatment of dyspepsia, urinary, and general debility diseases [8]. The whole plant of *T. cordifolia* has medicinal value; stems were reported useful in the treatments of fever, flatulence, diabetes, hypertension and leucorrhea [9,10]; roots were reported to be useful for spleen and gynecological disorders [11,12]. The starch of roots and stem was reported to be nutritious and used to cure chronic diarrhea and dysentery [13]. It is also known for its strong immunostimulant action due to the presence of a novel polysaccharide, α-d-glucan [14,15]. In addition, *T. cordifolia* is also a rich source of various micronutrients such as calcium, phosphorus, iron, zinc, copper and manganese [16]. The leaves of this plant are rich in protein (11.2%) and also calcium and phosphorus [17]. *T. cordifolia* has been studied for its antioxidant properties. In diabetic rats, the oral administration of aqueous *T. cordifolia* root extract lowered blood glucose levels [18]. The root, stem, and leaf extracts of *T. cordifolia* also show antimicrobial [19,20], antitoxic [21,22], antistress [23], anticancer [24,25,26], anti-HIV [27], and anti-osteoporotic [28,29] activities.

The survey of public databases and literature revealed extremely limited information with regard to molecular markers and SSRs in particular. SSR markers are very useful because of their codominant nature, their ability to detect multiallelic variability, and reliable reproducibility. These SSR markers have various applications in the field of genomics and are thus useful for the generation of high-density marker-based genetic maps for gene/QTL discovery, phylogenetic studies, and comparative genomics [30,31].

We have reported information on the transcriptome sequencing of *T. cordifolia* and also validated nearly 96 SSR markers across different *T. cordifolia* species [32,33]. Such research encouraged us to develop a dedicated database of transcriptome-based SSR markers along with annotation details of transcripts for scaling up research by the scientific community engaged in the genetic improvement of this medicinal plant. In the present study, we report a database of SSR markers and transcription factors (TinoTranscriptDB) and, to the best of our knowledge, this is the first relational database of transcriptome-based microsatellite makers and transcription factors that contains information of primer sequences along with the functional annotation details of respective transcripts. We also incorporated records of transcript sequences, blast results, gene ontology, motif details, and enzyme commission number details in the database so that users can access annotated transcript details for their intended use.

## 2. Materials and Methods

### 2.1. Raw Data Processing and De Novo Meta-Transcriptome Assembly

Our in-house leaf tissue sequenced RNA-Seq data of *T. cordifolia* generated through Roche-454 GS-FLX platform (SRA Acc. No. SRP018583), and another publicly available Illumina platform (SRA Acc. No. SRR15221490) was used in this study. The transcriptome shotgun assembly (TSA) of Roche 454 dataset (25,406 transcripts) was downloaded from the NCBI TSA database and Illumina SRA reads accession no. SRR15221490 was downloaded from the NCBI SRA database. Illumina raw reads were quality-checked using FASTQC (accessed on 2 June 2022; http://www.bioinformatics.bbsrc.ac.uk/projects/fastqc), and the adapters and poor-quality bases were trimmed using Trimmomatic V3.39 [34]. Then, filtered reads were assembled using rnaSPAdes software with default parameters [35]. The Illumina assembled transcripts (34,027) were further merged with our in-house de novo assembled transcripts (25,406) to generate a non-redundant meta-transcriptome assembly (43,090 transcripts) using the CH-HIT program with 90% identity [36]. This meta-transcriptome assembly was further used for downstream analysis.

### 2.2. In Silico SSR Mining and Primer Designing

The Krait v1.3.3 software (accessed on 17 June 2022; https://github.com/lmdu/krait) [37] was used to identify the microsatellite markers (SSRs) in the non-redundant assembled transcripts dataset using the search criteria of six repeat units for di-nucleotide and five repeat units for tri-, tetra-, penta- and hexa-nucleotide repeats and the maximal number of bases interrupting 2 SSRs in a compound microsatellite to be 100 bp. The output results of Krait contained information such as repeat number, motif sequence, length of SSR repeat, type of repeats, start and end positions, Tm, and GC content. The primer pairs were designed from the 200 bp flanking regions of SSR motifs using Primer3 v1.0 (https://github.com/primer3-org/primer3, accessed on 17 June 2022) [38] with parameters (primer length = 20–25 bp; size of PCR product = 100–250 bp, with optimum of 280 bp; annealing temperature = 65 °C; GC content of 40–60% with optimum of 50%).

### 2.3. Functional Annotation of Transcripts

The non-redundant transcript dataset was BLAST-searched against different databases including NCBI NR, Swiss-Prot, Pfam, gene ontology (GO), and KEGG using BLASTX program with a cut-off E-value of 1 × 10^−10^ [39]. The functional descriptions were assigned to non-redundant transcripts with BLAST search against NCBI non-redundant and Swiss-Prot protein databases using BLAST2GO 6.0.3 [40] followed by a search for gene ontology terms (GO), to carry out pathway analysis (using KEGG), and assign enzyme commission (EC) numbers.

### 2.4. Identification of Transcription Factors and Gene Families

The transcription factors (TFs), working as molecular switches, were identified using the entire non-redundant assembled transcript set, and were BLAST-search against the Plant Transcription Factor Databases (PlantTFDB v4.0) [41] with a parameters bit score > 100 and e-value of 1 × 10^−3^ [42]. The annotation of the identified TFs was performed using InterProScan ver.5.56 [43], which contains various inbuilt functional databases including Pfam (http://pfam.xfam.org/, accessed on 17 June 2022), PRINTS (http://www.bioinf.manchester.ac.uk/dbbrowser/PRINTS/, accessed on 17 June 2022), SMART (http://smart.embl-heidelberg.de/, accessed on 17 June 2022), PrositePatterns (http://prosite.expasy.org/, accessed on 17 June 2022), PrositeProfiles (http://prosite.expasy.org/, accessed on 17 June 2022), SUPERFAMILY (http://supfam.cs.bris.ac.uk/, accessed on 17 June 2022), Panther (http://www.pantherdb.org/panther/, accessed on 17 June 2022) and gene ontology (GO) (http://amigo.geneontology.org/, accessed on 17 June 2022), to predict the functional domains and structural motifs along with their annotation details.

The gene families related to the biosynthesis of biochemical compounds in *T. cordifolia* were also analyzed based on annotation of transcripts.

### 2.5. Database Architecture and Web Interface

**Figure 1:**
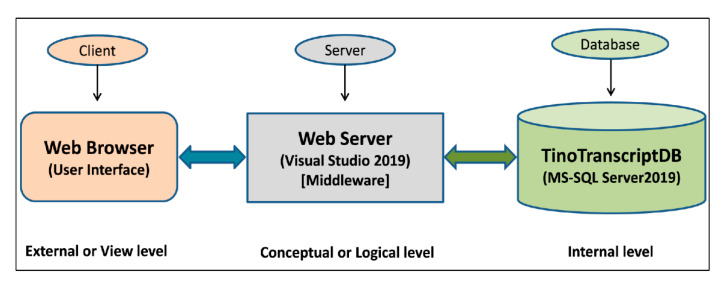
Three-level database schema architecture used for the construction of TinoTranscriptDB.

**Figure 2:**
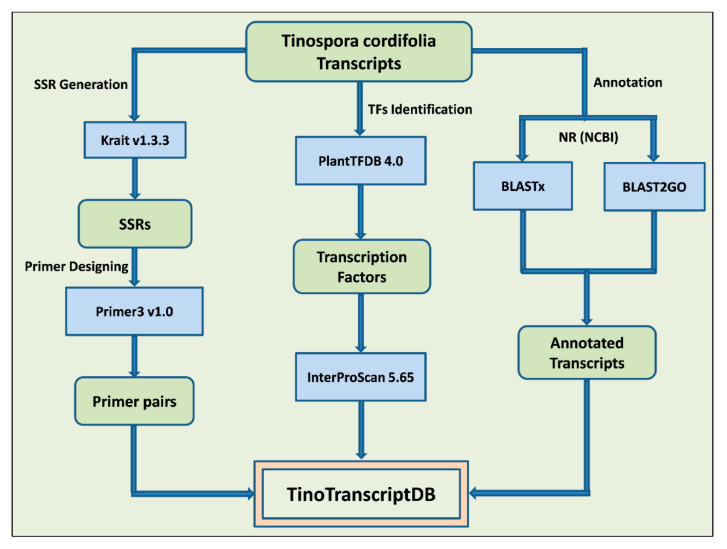
Process flow diagram and tools used for the transcriptome SSR marker identification, primer designing, annotation and transcription factor categorization.

*T. cordifolia* transcripts and SSR marker database (TinoTranscriptDB) is a relational and interactive database that contains detailed information on annotated transcripts, SSR markers with three pairs of primers, and TFs with their functional annotation details. Additionally, it also contains transcripts’ sequence information from which SSR markers were designed. This database is based on “three level schema architecture” with client tier, server tier, and database tier (Figure 1). The interactive user interface of the database was designed and developed using a server-side web programming language (ASP.NET), and database tables were stored in MSSQL Server 2019. The schematic of the data generation for the present database creation is shown in Figure 2.

## 3. Results

### 3.1. Microsatellite Markers and Primer Pairs

From the non-redundant assembled transcripts dataset, a total of 7980 perfect SSRs, 360 compound SSRs, and 32,551 imperfect SSRs were identified (Table S1). Here, our main focus was perfect SSRs because perfect SSRs are more useful as compared to compound and imperfect SSRs. Out of the total perfect SSRs, tri-nucleotides were the most abundant at 50.13% (32,007), followed by di-nucleotides at 41.95% (24,158), tetra-nucleotides at 4.50% (3588), penta-nucleotides at 2.98% (3075), and hexa-nucleotides at 0.44% (528) (Figure S1). Mono-nucleotides were excluded from our analysis. Among the different types of repeat motifs, it was observed that in each motif category, one particular motif category was dominant. Among all the identified SSR motifs of the total di-nucleotide repeats, 9.52% were “AT”, 8.86% of the total tri-nucleotide repeats were “AAG”, 0.83% of the total tetra-nucleotide repeats were “AAAT”, 0.81% of the total penta-nucleotide repeats were “AATGG”, and 0.01% of the total hexa-nucleotide repeats were “AAAAAC” (Table S2). Out of the total identified SSRs, three primer pair sequences were generated for 2620 perfect, 136 compound, and 18,272 imperfect SSRs from 2200, 135, and 12,099 transcripts, respectively. Among the three primer pairs of 2620 perfect SSRs, tri-nucleotide repeat primer pairs were the most abundant (54.31%), followed by di-(37.51%), tetra-(4.54%), penta-(3.16%), and hexa-nucleotides (0.45%) (Table 1).

### 3.2. Transcripts Annotation

**Figure 3:**
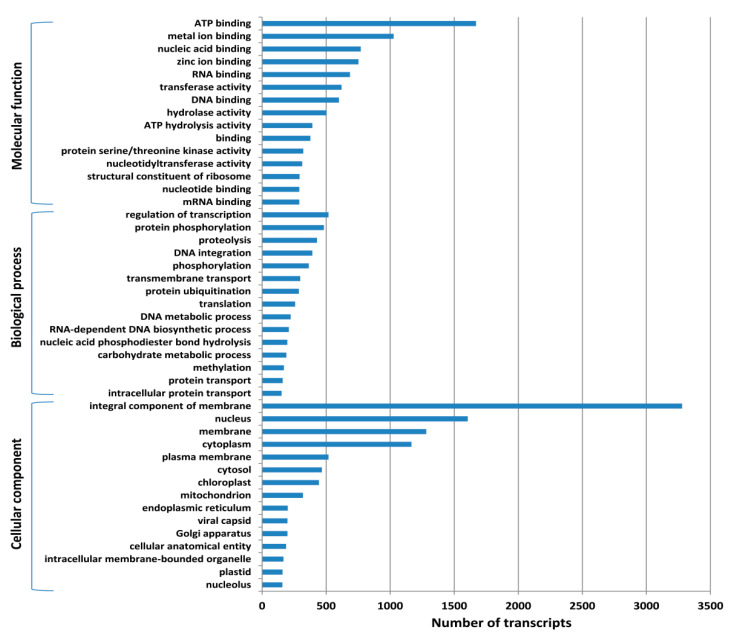
Functional classification of transcripts based on GO terms, distributed in three major categories: molecular function, biological process, and cellular component.

In order to determine the function of transcripts, we blasted all 43,090 transcript sequences against the non-redundant (nr) protein database (NCBI) using the BLASTx program with an E-value cut off of 1 × 10^−10^. A total of 26,487 (61.47%) transcripts showed significant similarity with the NCBI-NR protein database. The similarity distribution of the BLAST hits showed that more than 50% of the transcript sequence had similarities in the range of 40% to 100% (Figure S2). The species distribution graph of the BLAST results showed the highest similarity of *T. cordifolia* transcripts against *Vitis vinifera*, followed by *Nelumbo nucifera*, *Aquilegia coerulea*, and other crop species (Figure S3). Further, 16,614 (38.55%) of the total transcripts showed significant matches with the Swiss-prot database (Table S3). Out of the 26,487 transcripts, 20,364 transcripts were assigned GO terms. A total of 84,172 GO terms were assigned to 20,364 transcripts and their distribution was broadly categorized into molecular functions, biological processes and cellular components (Table S4). Based on the assigned GO terms, the maximum number of transcripts were found to be in “ATP binding” and “metal ion binding” under molecular function category; “regulation of transcription” and “protein phosphorylation” under the biological process category; and “integral component of membrane” and “nucleus” under the cellular component category (Figure 3). A total of 9,259 transcripts were assigned enzyme codes and enzyme names. Out of these, the maximum number of transcripts encode translocases followed by transferases, hydrolases, and oxidoreductases (Table S5).

### 3.3. Transcription Factors

**Figure 4:**
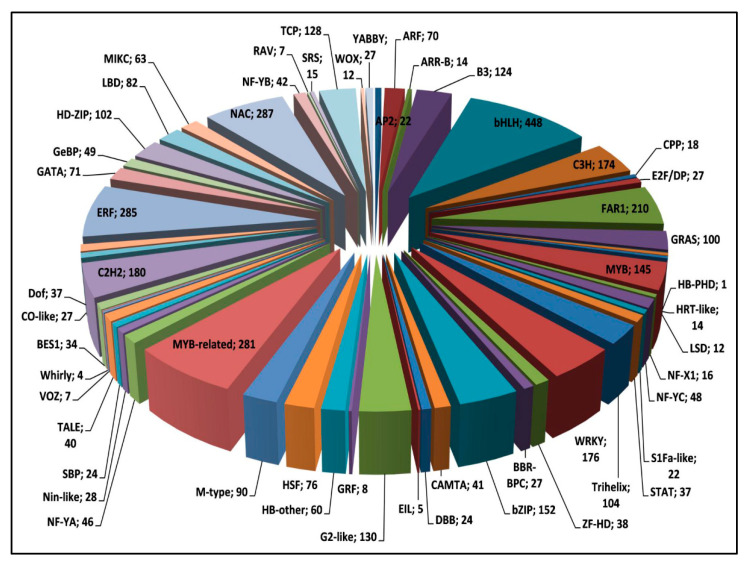
Pie-chart distribution of *T. cordifolia* 4311 transcripts in to 55 TF categories based on the BlastX search against the PlantTFDB.

**Figure 5:**
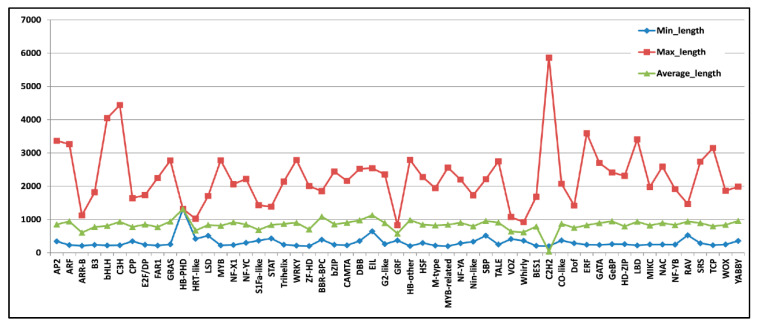
Line graph coverage graph of identified 4311 *T. cordifolia* transcripts categorized into 55 different TF categories based on minimum, maximum and average transcript length of each TFs category.

A total of 4311 TFs were identified and were distributed among 55 TF families (Figure 4). Among the TF families, the maximum number of TFs were included in bHLH (448), followed by NAC (287), ERF (285), MYB (281), and FAR1 (210) (Table S6). Interestingly, among various TFs families, the length of the assembled transcript of different TFs, ranged between ~200 and 5865 bp (Figure 5). As the lengths of the majority of the assembled transcripts of TFs are in a workable range in terms of designing primer pairs, we can explore the spatial/temporal expression of candidate TFs along with other assembled transcripts of comparable length. The annotation of 4311 TFs using Inter ProScan showed significant matches with different databases such as pfam, PRINTS, Prosite Pattern, Prosite Profiles, Gene3D, and Panther (Table S7).

### 3.4. Gene Families Identified for Biochemical Synthesis

The annotated transcripts were analyzed to identify the gene families involved in the biochemical synthesis of compounds having medicinal value such as, cytochrome p50, kinases, HSPs, and transporters. The cytochrome p50 represents one of the largest enzyme families which play a crucial role the biosynthesis of secondary metabolites, antioxidants biosynthesis, fatty acid metabolism, xenobiotic metabolism, hormone regulation, and plant defense in higher plants [44]. In the present database, 122 transcripts have been identified to be related to cytochrome p50 (Table S8). Similarly, 510 transcripts have been identified as related to protein kinases, which play a fundamental role in signal transduction pathways, regulating a number of cellular functions such as cell growth, differentiation, and cell death. A number of plant extracts and their isolated secondary metabolites such as flavonoids, phenolics, terpenoids, and alkaloids have exhibited activities against various kinases [45]. A total of 30 transcripts related HSPs were found and are known to function as molecular chaperones, which are involved in the therapeutic treatments of many diseases [46]. Transporters are involved in the transport of plant secondary metabolites and a total of 257 transcripts related to transporter genes were found (Table S8). The transporters are used for the transport of secondary metabolites, such as alkaloids, terpenoids, phenolics, steroids, glycosides, aliphatic compounds and polysaccharides [47].

### 3.5. Database Utilities

We designed an interactive and user-friendly online database of transcript sequences, which contains information of various categories of TFs, SSR markers and GO components. The TinoTranscriptDB was developed to provide uninterrupted public access and deliver useful information about an important medicinal plant, *T. cordifolia*. TinoTranscriptDB has a user-friendly interface for efficient access, visualization and retrieval of information based on queries of interest with a simple or combination of search criteria along with a useful data download option. In the TinoTranscriptDB, data are organized into three major sections: (1) SSR markers with their three pairs of primer details, (2) transcription factors with their annotation information, and (3) annotated transcripts categories based on GO numbers. Using various search options, users can access the database to browse and download various structural (SSR details) and functional (annotation details) features of the *T. cordifolia* transcriptome.

**Figure 6:**
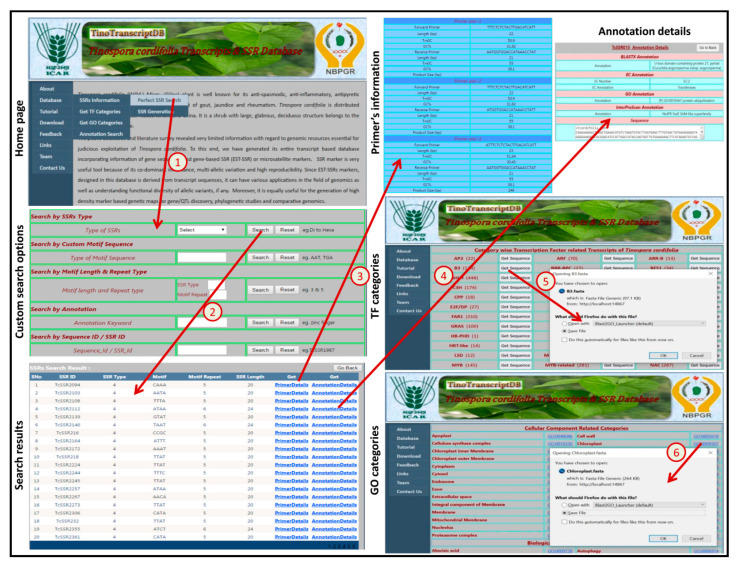
Screenshot of the TinoTranscriptDB showing SSR and primer searching using different search options, (1) arrow shows perfect SSR search criteria on SSR search page; (2) arrow shows search for SSR di to hexa types; (3) arrow shows Primer Details link of particular SSR; (4) arrow shows Annotation Details link of particular SSR with annotation information; (5) arrow shows Sequences link of that TF category in a fasta sequence file; (6) arrow shows GO Id in fasta sequence file.

**Figure 7:**
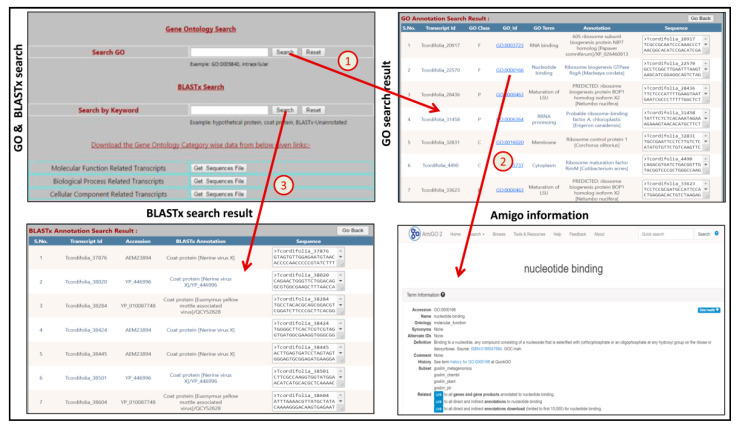
Screenshot of the TinoTranscriptDB showing transcript annotation searching with the download option for several sequences, (1) arrow shows Gene ontology search by the GO Id; (2) arrow shows by click on the particular GO Id detailed information on a separate page; (3) arrow shows BLASTX search by the annotation keyword on a separate page.

TinoTranscriptDB has nine different tabs with six sub-tabs under database tab (About, Database, BLAST, Tutorial, Download, Feedback, Links, Team and Contact Us) (Figure 6). The analysis summary of the transcript dataset is shown under the SSR generation tab and the tutorial page contains detailed information regarding the usage of the database. In the TinoTranscriptDB, we have highlighted perfect SSRs because perfect SSRs are more useful as compared to compound and imperfect SSRs. An SSR search of *T. cordifolia* is based on SSR ID, SSRs type, motif sequence, annotation keyword or both motif type and repeats (Figure 6). The search result page provides basic information about the SSR markers, such as SSR ID, SSR type, SSR motif, motif repeat, and SSR length, along with two hyperlinked fields, one for primer details and another for annotation details. When users click on “primer details”, the link takes them to the next web page which displays information related to three sets of primer pairs (Figure 6). Similarly, clicking the “annotation details” takes users to the next web page which contains the full annotation details, including information on gene ontology, Pfam annotation, enzyme commission (EC) number, and transcript sequence from which the SSR has been identified (Figure 6). This annotation result will help researchers in selecting transcripts of their interest for designing primer pairs. The TF categories tab can be used to obtain category-wise TF-related transcripts in Fasta format (Figure 6). Similarly, from the GO categories tab, users can obtain GO category-wise transcripts in Fasta format (Figure 6). In GO categories, entire assembled transcripts were grouped into three major categories as cellular components, biological processes, and molecular function. Each category has subgroups as well as respective transcript information. These Fasta sequences of TF-related as well as GO-related transcripts may be used for further downstream analysis. TinoTranscriptDB also has a separate search function for BLASTx as well as gene ontology (GO) annotation, from which users can view annotations, accession or ID, GO class, and transcript sequences as per the query fed in functional keywords or GO number search functions (Figure 7). From this page, users can also download the transcript data, categorized into three major GO categories (cellular component, molecular function and biological process) in separate Fasta files. The local BLAST feature of TinoTranscriptDB enables the user to compare query sequence with the *T. cordifolia* transcripts.

## 4. Discussion

For the strategic improvement of the genetic traits of germplasm resources, more specifically, for enhancing their economic potential, there is a prior requirement of information related to genomic resources. Such information includes not only sequences pertaining to the genetic determinants that regulate the outcome of the phenotype (functional resources), but also information related to molecular markers (structural resources). Molecular markers serve as mileposts for the genome as they are distributed randomly genome-wide but with fixed locations. Among several molecular markers, the microsatellites, also known as simple sequence repeats (SSR), have become more essential for speeding up research not only through molecular breeding approaches but also in the area of the diversity assessment of the germplasms of plant species. Moreover, if such SSRs are developed based on the sequence information of transcripts, the utilities of such markers can be expanded further. Such gene-based SSR markers are also useful for determining candidate genes, exploring allelic variation, etc. In the development of a database of gene-based SSR markers, utilizing the sequence information of transcripts is researchers a promising method to expand research in the area of marker-assisted selection aimed toward the genetic enhancement of *T. cordifolia*. Additionally, such markers can also be exploited to assess diversity in the available germplasm of *T. cordifolia* [32]. The SSRs can also be employed in related species of *T. cordifolia* through transferability studies [33]. We have already tested the efficacy of transferability studies in related species. The annotation details of transcripts, from which SSRs have been developed, are useful for researchers to help them select markers. The allelic variation with respect to any SSR marker can also provide information related to changes in phenotype, if any. In addition to information on SSR markers, the database also includes annotation details of assembled transcripts, including those of TFs and gene families related to biochemical compound synthesis with medicinal values, thus offering researchers a choice to clone their favorite genes associated with the phenotype of interest.

## 5. Conclusions

TinoTranscriptDB is a user-friendly database, accessible freely via the address http://www.nbpgr.ernet.in:8080/Tinospora/ (accessed on 17 June 2022), contains a total of 2620 SSR markers with three primer pairs for each identified SSR marker. This database provides users with the flexibility to leverage not only information on SSRs, but also information about transcripts of *T. cordifolia*. To facilitate the usage of transcript-related information, the entire transcripts were categorized into three GO functional groups, TF categories, and gene families. This database is expected to be of practical significance to researchers working on the genetic improvement of traits of economic importance in *T. cordifolia*. In addition, this database will facilitate molecular breeding programs and other applied studies in *T. cordifolia* and related medicinal plant species.

## Figures and Tables

**Table 1:** Distribution of SSR motifs (di to hexa) with three primer pairs, their relative abundance (loci/Mb) and relative density (bp/Mb).

SSR Type	No. of SSRs	Length (bp)	Percentage (%)	Relative Abundance (loci/Mb)	Relative Density (bp/Mb)
Di	983	15,394	37.51	41.90	656.2
Tri	1423	26,262	54.31	60.66	1119.4
Tetra	119	2744	4.54	5.07	117.0
Penta	83	2500	3.16	3.54	106.6
Hexa	12	408	0.45	0.51	17.4

## Data Availability

All the data and Supplementary Materials are available at database online.

## Supplementary Materials

The following supporting information can be downloaded at: https://www.mdpi.com/article/10.3390/genes13081433/s1, Figure S1: Percentage distribution of di to hexa type of perfect SSRs; Table S2: Abundance of each type of SSR motifs in di to hexa categories; Figure S2: Sequence similarity distribution graph of BLAST hits; Figure S3: Sequence similarity distribution of *T. cordifolia* transcripts with other species; Table S1: The summary of Perfect, Compound, and Inperfect Microsatellite marker types; Table S2: Abundance of each type of SSR motif category; Table S3: Transcripts similarity distribution with SwissProt database; Table S4: Gene Ontology (GO) assignment of the transcripts; Table S5: Enzayme codes and Enzyme name assignments of the transcripts; Table S6: TF categories their maximum, minimum and average sequence lengths; Table S7: Transcripts annotation with InterProScan; Table S8: Transcripts categorized into gene families related to the biosynthesis of biochemical compounds in *T. cordifolia*.
