# Supplementary figures and images for: TinoTranscriptDB: A Database of Transcripts and Microsatellite Markers of Tinospora cordifolia, an Important Medicinal Plant
Source: Genes (Basel). 2022 Aug 12;13(8):1433. doi: 10.3390/genes13081433 (PMC9407948; doi:10.3390/genes13081433)

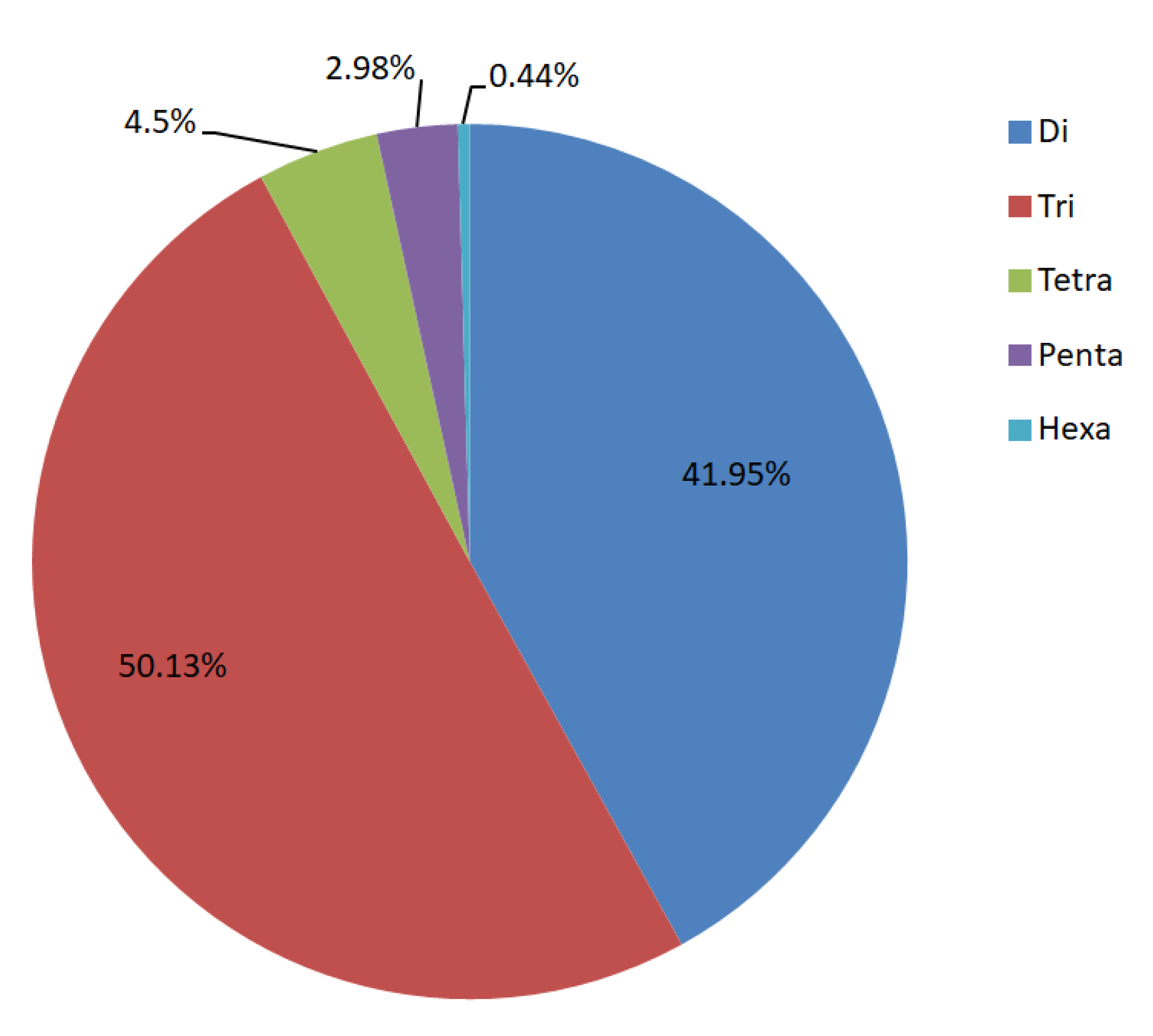

Supplement: Supplementary file 1 [file genes-13-01433-s001.zip › Figure S1.tif]

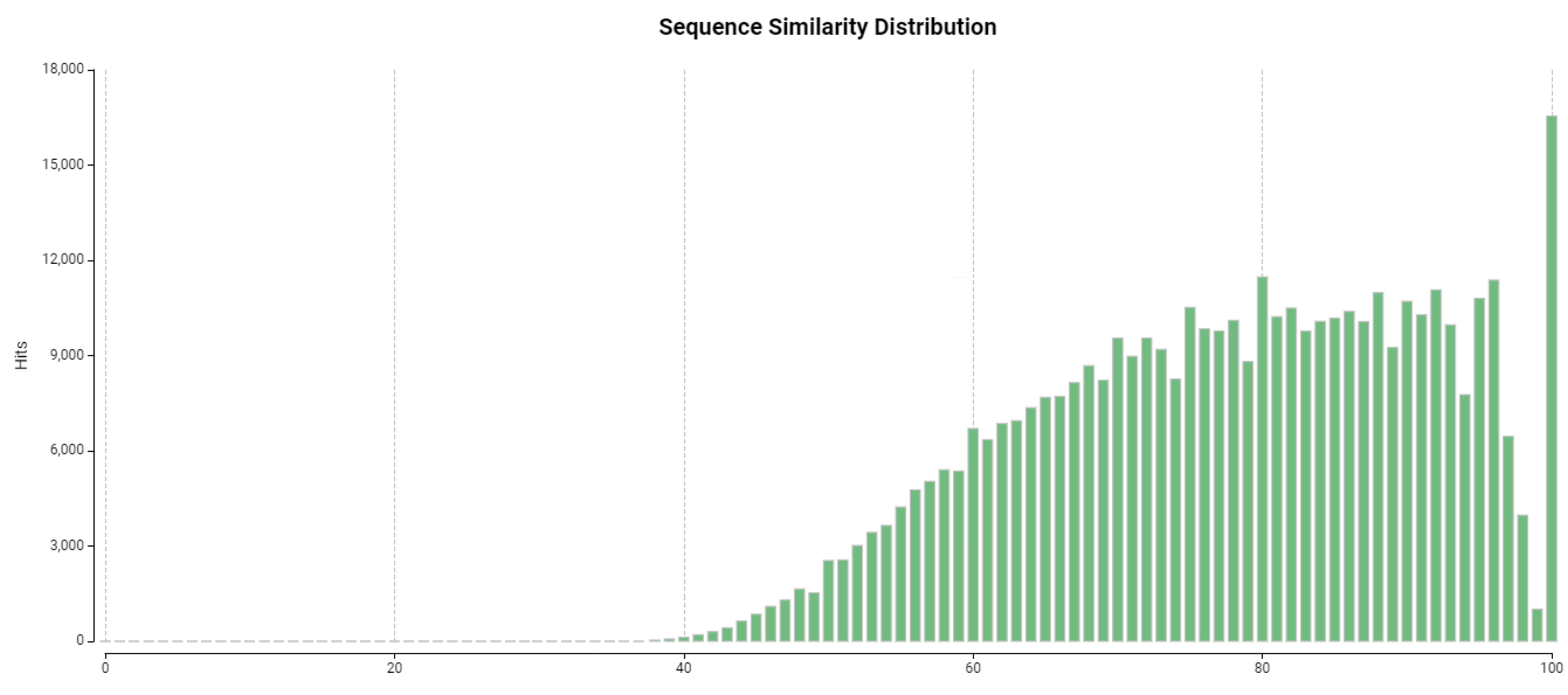

Supplement: Supplementary file 1 [file genes-13-01433-s001.zip › Figure S2.tif]

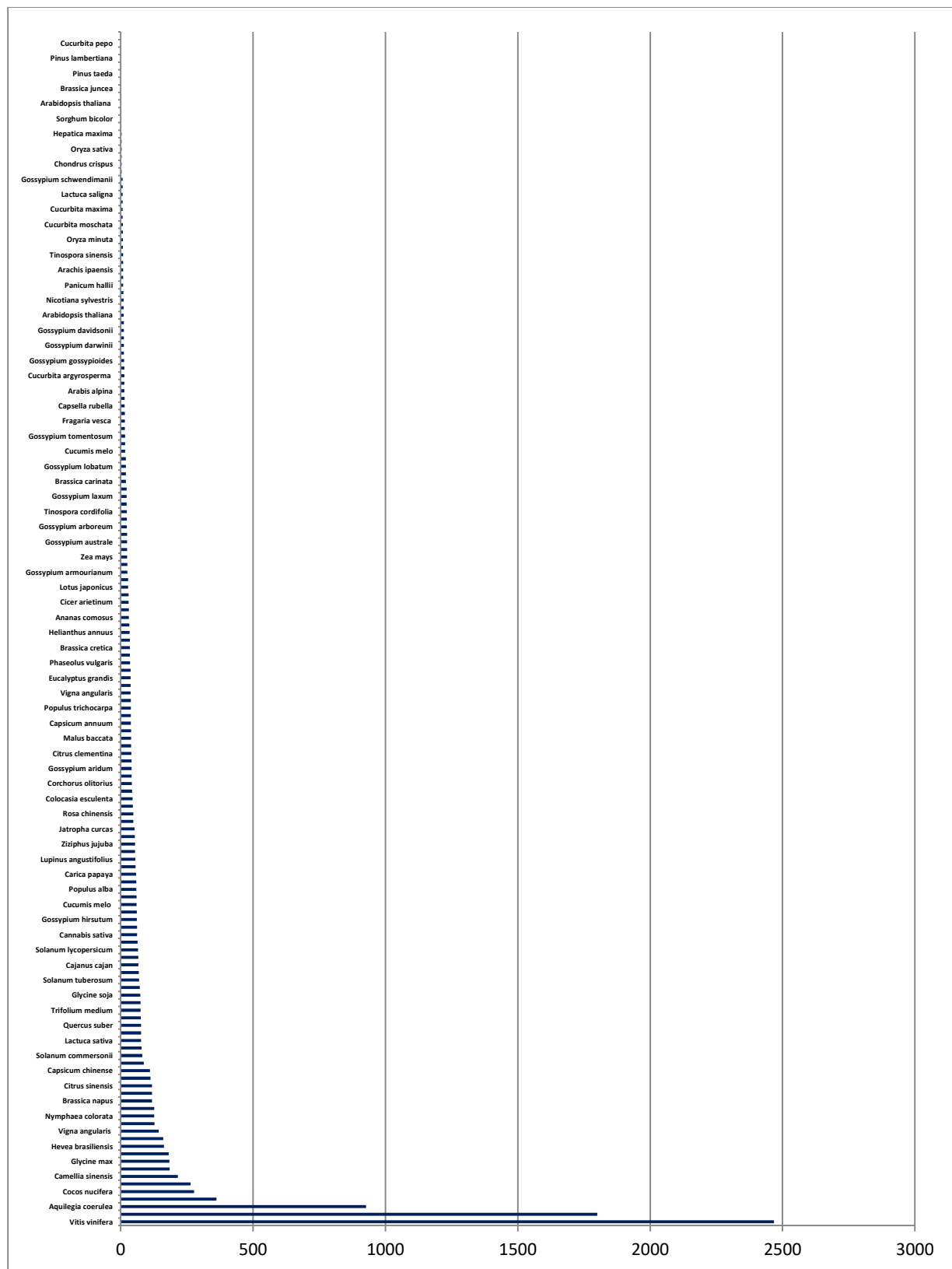

**Figure S3.** Sequence similarity of *T. cordifolia* transcripts with other species.

Supplement: Supplementary file 1 [file genes-13-01433-s001.zip › Figure S3.pdf]
